# Histotripsy for the Treatment of Cholangiocarcinoma Liver Tumors: *In Vivo* Feasibility and *Ex Vivo* Dosimetry Study

**DOI:** 10.1109/TUFFC.2021.3073563

**Published:** 2021-08-27

**Authors:** Alissa Hendricks-Wenger, Peter Weber, Alex Simon, Sofie Saunier, Sheryl Coutermarsh-Ott, Douglas Grider, Joan Vidal-Jové, Irving Coy Allen, David Luyimbazi, Eli Vlaisavljevich

**Affiliations:** Department of Biomedical Engineering and Mechanics, Virginia Tech, Blacksburg, VA 24061 USA, also with the Department of Biomedical Sciences and Pathobiology, Virginia-Maryland College of Veterinary Medicine, Blacksburg, VA 24061 USA, and also with the Graduate Program in Translational Biology, Medicine and Health, Virginia Tech, Roanoke, VA 24016 USA; Virginia Tech Carilion School of Medicine, Roanoke, VA 24016 USA; Department of Biomedical Engineering and Mechanics, Virginia Tech, Blacksburg, VA 24061 USA; Department of Biological Systems Engineering, Virginia Tech, Blacksburg, VA 24061 USA; Department of Biomedical Sciences and Pathobiology, Virginia-Maryland College of Veterinary Medicine, Blacksburg, VA 24061 USA; Department of Basic Science Education, Virginia Tech Carilion School of Medicine, Roanoke, VA 24016 USA, and also with Dominion Pathology Associates, Roanoke, VA 24016 USA; Institute Khuab for Interventional Oncology, Comprehensive Tumor Center Barcelona, 08023 Barcelona, Spain; Department of Biomedical Sciences and Pathobiology, Virginia-Maryland College of Veterinary Medicine, Blacksburg, VA 24061 USA, also with the Graduate Program in Translational Biology, Medicine and Health, Virginia Tech, Roanoke, VA 24016 USA, and also with the Department of Basic Science Education, Virginia Tech Carilion School of Medicine, Roanoke, VA 24016 USA; Virginia Tech Carilion School of Medicine, Roanoke, VA 24016 USA, and also with the Department of Surgery, Carilion Clinic, Roanoke, VA 24016 USA; Department of Biomedical Engineering and Mechanics, Virginia Tech, Blacksburg, VA 24061 USA, and also with the Graduate Program in Translational Biology, Medicine and Health, Virginia Tech, Roanoke, VA 24016 USA

**Keywords:** Medical imaging, medical transducers, system & device design, therapeutics

## Abstract

Histotripsy is a noninvasive, nonionizing, and nonthermal focused ultrasound ablation method that is currently being developed for the treatment of liver cancer. Promisingly, histotripsy has been shown for ablating primary [hepatocellular carcinoma (HCC)] and metastatic [colorectal liver metastasis (CLM)] liver tumors in preclinical and early clinical studies. The feasibility of treating cholangiocarcinoma (CC), a less common primary liver tumor that arises from the bile ducts, has not been explored previously. Given that prior work has established that histotripsy susceptibility is based on tissue mechanical properties, there is a need to explore histotripsy as a treatment for CC due to its dense fibrotic stromal components. In this work, we first investigated the feasibility of histotripsy for ablating CC tumors *in vivo* in a patient-derived xenograft mouse model. The results showed that histotripsy could generate CC tumor ablation using a 1-MHz small animal histotripsy system with treatment doses of 250, 500, and 1000 pulses/point. The second set of experiments compared the histotripsy doses required to ablate CC tumors to HCC and CLM tumors *ex vivo*. For this, human tumor samples were harvested after surgery and treated *ex vivo* with a 700-kHz clinical histotripsy transducer. Results demonstrated that significantly higher treatment doses were required to ablate CC and CLM tumors compared to HCC, with the highest treatment dose required for CC tumors. Overall, the results of this study suggest that histotripsy has the potential to be used for the ablation of CC tumors while also highlighting the need for tumor-specific treatment strategies.

## I. Introduction

MORE than 800 000 people are diagnosed with a malignancy originating from or metastatic to the liver every year. Of these, 42 810 cases are projected for the U.S. in 2020 [1]. These malignant liver tumors include hepatocellular carcinoma (HCC), liver metastasis (LM), and cholangiocarcinoma (CC). Although surgical resection remains the mainstay of liver tumor intervention, less than 25% of patients are candidates for resection due to tumor burden, location, or comorbid disease [2], [3]. For example, a prior study found that only 15% of intrahepatic CC tumors and 56% of distal tumors were deemed resectable [4].

Minimally invasive ablation methods have shown some success in treating liver tumors but have limitations due to the thermal mechanism, including lack of precision and high complication rates when treating near critical structures [5]–[14]. Histotripsy is a noninvasive, nonthermal, and image-guided focused ultrasound ablation method that ablates tissue through the generation of acoustic cavitation at the transducer focus [15]–[19]. Histotripsy uses microsecond long, high-pressure pulses to generate a histotripsy bubble cloud inside of the targeted tissue [20]–[24]. The rapid expansion and collapse of the cavitation bubbles result in complete disruption of the tissue [25]. As a nonthermal ablation modality, histotripsy has shown the potential to overcome the limitations associated with thermal ablation. For instance, hepatic histotripsy has been shown to produce consistent and complete ablation in highly vascular regions of the liver [18], [26]. Histotripsy has also shown the ability to preserve vital structures, such as major vessels, bile ducts, and nerves within the ablation zone [18], [27]. Preclinical *in vivo* studies of hepatic histotripsy in healthy porcine and rodent models have demonstrated that histotripsy can generate clinically relevant ablation zones with sharp boundaries (<1 mm) between treated and healthy liver tissue [15], [17], [18], [26], [27]. Additional studies have shown the *in vivo* feasibility of treating HCC tumors in rodent models using histotripsy [19], [28]. Based on these successful preclinical studies, a Phase I clinical trial in patients with liver cancer was recently conducted to demonstrate the feasibility of hepatic histotripsy [16]. Together, these studies have established the feasibility and overall safety profile of hepatic histotripsy.

Although one patient with CC was reportedly treated as part of the clinical trial mentioned above, there have been limited studies investigating the potential of histotripsy for treating CC tumors. CC tumors pose a unique challenge for ablation modalities due to their common location near vital critical structures [29]. The vessel and duct sparing features of hepatic histotripsy are promising for the treatment of CC located near these structures. However, given that prior studies showing fibrous tissues with higher stiffness and mechanical strength are more resistant to histotripsy, it is also expected that CC tumors may pose a unique challenge for histotripsy. This hypothesis is based on the CC tumor’s dense desmoplastic fibrotic composition and higher stiffness that have been reported previously [24], [30], [31]. For instance, prior work has found the average Young’s modulus of CC tumors to be 56.9 ± 25.6 kPa, which was significantly higher than HCC (14.86 ± 10.0 kPa) and LM (28.8 ± 16.0 kPa) tumors [32]. Given these mechanical profiles, we hypothesize that histotripsy will require higher treatment doses to ablate CC tumors compared to LM and HCC.

In this study, we investigate the feasibility of using histotripsy for the treatment of CC tumors. To test histotripsy for CC ablation, this study was split into two parts. In part one, we investigate the *in vivo* feasibility of histotripsy CC ablation in a subcutaneous patient-derived xenograft (PDX) CC tumor model. In part two, we perform a comparative dosimetry study for treating *ex vivo* human HCC, LM, and CC tumors with histotripsy to determine the differences in treatment dose requirements for ablating each tumor type with histotripsy.

## II. Procedures

### A. Histotripsy Systems

**Fig. 1.**
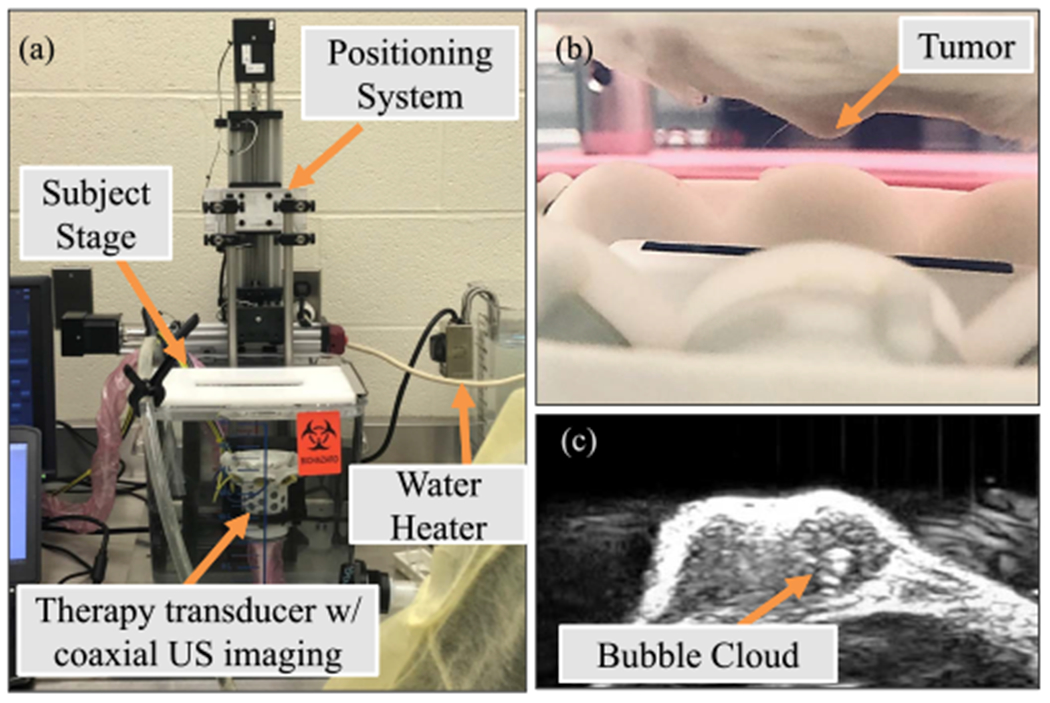
*In vivo* ablation setup. (a) Histotripsy murine experiments were conducted using a custom 1-MHz small animal histotripsy system. (b) Subject’s tumors were submerged in degassed water at the transducer focus in order for (c) histotripsy to be applied to the tumor noninvasively guided by real-time ultrasound imaging.

Two separate histotripsy systems were used in this study for the small animal and large animal *in vivo* histotripsy studies. Small animal *in vivo* experiments were conducted using a custom eight-element 1-MHz histotripsy transducer with a geometric focal distance of 36 mm, an aperture size of 52.7 mm, and an f-number of 0.68 [see Fig. 1(b)]. The transducer was driven via a custom high-voltage pulser designed to generate short therapy pulses of less than two cycles controlled by a field-programmable gate array (FPGA) board (Altera DE0-Nano Terasic Technology, Dover, DE, USA) programed for histotripsy therapy pulsing. The transducer was positioned in a tank of degassed water beneath a custom-designed mouse surgical stage (see Fig. 1) and attached to a computer-guided 3-D positioning system with a 0.05-mm motor resolution to control the automated volumetric treatments. A linear ultrasound imaging probe with a frequency range of 10–18 MHz (L18-10L30H-4, Telemed, Lithuania, Europe) was coaxially aligned inside the transducer for treatment guidance and monitoring [19], [28]. The transducer was powered by a high voltage dc power supply (GENH750W, TDK-Lambda), and the system was controlled using a custom user interface operated through MATLAB (MathWorks).

For the *ex vivo* experiments, histotripsy was applied using a 36-element 700-kHz clinical histotripsy transducer and associated driving systems (HistoSonics, Inc.) that was previously used in a human clinical trial for the treatment of benign prostatic hyperplasia [33] and multiple previous preclinical studies [24], [34]. This system is the same frequency as the clinical histotripsy system used in the recent Phase I human liver cancer trial [16] and was, thus, chosen for this study. The transducer consists of a concave circular aperture with a small inlet on the perimeter to allow for histotripsy treatments to be guided by a transrectal imaging probe (note that this imaging probe was not used in this study [24], [34]; the transducer has a geometric focal distance of 11 cm and full-width half-maximum (FWHM) dimensions at a geometric focus of 4.0, 4.0, and 15.0 mm in the transverse, elevational, and axial dimensions, respectively. The transducer was powered by a high-voltage dc power supply (GENH750W, TDK-Lambda), and the system was controlled using a custom user interface called the Histotripsy Service Tool (HistoSonics, Inc.) to produce five-cycle histotripsy pulses [24], [35].

### B. Pressure Calibration

Focal pressure waveforms for the 1-MHz and 700-kHz transducers were measured by a high sensitivity rod hydrophone (HNR-0500, Onda Corporation, Sunnyvale, CA, USA) and a custom-built fiber optic probe hydrophone (FOPH) [36], [37] in degassed water at the focal point of each transducer. Focal pressures were directly measured with the FOPH up to a peak negative pressure (*p*-) of ~16 MPa for the 700-kHz transducer and ~20 MPa for the 1-MHz transducer. Pressures were not directly measured beyond these limits due to cavitation on the tip of the hydrophone probe. Waveforms at higher *p*- were estimated using the linear summation of the pressure waveforms directly measured from four subapertures of the array for the 700-kHz transducer and two subapertures for the 1-MHz transducer. This method showed close agreement between directly measured pressures and the cavitation threshold seen experimentally [38]. All waveforms were measured using a Tektronix TBS2000 series oscilloscope at a sample rate of 500 MS/s, with the waveforms averaged over 128 pulses and recorded in MATLAB.

### C. Histotripsy in Vivo CC Ablation Procedure

The feasibility of histotripsy for CC tumor ablation was tested *in vivo* using a PDX CC mouse model. Twelve female NSG mice with subcutaneous flank PDX CC tumors were ordered from and prepared by Jackson Laboratory (Bar Harbor, ME, USA). Through a process previously described for pancreatic PDX tumors [39], a human CC tumor was collected from a patient and propagated as a heterogeneous tumor in immune-compromised NSG mice. Once received, animals were monitored three times weekly under Virginia Tech IACUC protocols. At each check, general health was monitored, and the tumor diameter was determined by taking two perpendicular measurements, one of which was the widest point of the tumor. Single tumor diameter was calculated as the square root of the product of the two measurements. The mice underwent histotripsy treatment when their tumors reach the targeted treatment size of ~5-mm diameter. Before treatment, fur was removed over the tumor and the surrounding area by applying Nair (Naircare, Ewing, NJ, USA) for 1–2 min and removed by wiping off with a wet paper towel. For treatment, mice were anesthetized with isoflurane (1.5-L/min oxygen flow rate with 1.0%−2.5% isoflurane) and placed prone on the subject stage (see Fig. 1) with their tumor submerged underwater. Animals’ respiratory rate and rhythm were monitored during treatment by trained personnel.

The overall setup for the *in vivo* treatment is illustrated in Fig. 1. This custom system, which has been used in previous small animal *in vivo* histotripsy studies [17], [19], [28], consists of the 1-MHz histotripsy transducer with coaxially aligned ultrasound imaging positioned vertically underneath a small animal surgical stage inside a heated water tank that allows for acoustic coupling. A second water tank is connected to this primary tank to circulate warmed, degassed water at a constant temperature throughout the procedures. Before treatment, the water tanks were filled with water and degassed for approximately 1 h. A water heater was used to maintain the water temperature at 37 °C + 4 °C throughout the treatment. A small animal platform containing a hole in the center was located on top of the tank for an anesthetized mouse to be placed with the mouse’s flank containing the tumor submerged in the water at the transducer focus. The transducer and a coaxially aligned imaging probe were connected to a 3-D motorized positioning system that was used to apply an automated volumetric ablation to a targeted volume within the tumor. This custom volumetric ablation system was designed to allow the user to outline the desired tumor and treatment regions before starting treatment. The treatment volume was then divided into a 3-D grid of points, and histotripsy was raster-scanned through a series of treatment points spaced by 1.5 mm in the axial direction and 0.5 mm in the lateral directions. While all the tumors were treated at different sizes due to delayed growth and planning in between treatments, the goal was to treat tumors when they averaged 5 mm in diameter. For a 5-mm-diameter tumor, the spacing resulted in treatment with 1382 treatment points, a dwell time of 2 s, and a 46-min treatment. This treatment spacing was determined based on preliminary experiments to ensure overlap between adjacent bubble clouds. Three separate treatment doses of 250, 500, and 1000 pulses/point were tested to determine the feasibility of histotripsy for treating CC tumors *in vivo*, with the results compared to an untreated group (*n* = 3/group). Treatment dose was determined based on prior small animal studies that utilized 50–500 pulses to treat healthy liver and HCC tumors in addition to the hypothesis that CC tumors would require a high dose given their higher mechanical strength [17], [19], [28].

### D. Human Liver Tumor Specimens for Ex Vivo Treatments

**Fig. 2.**
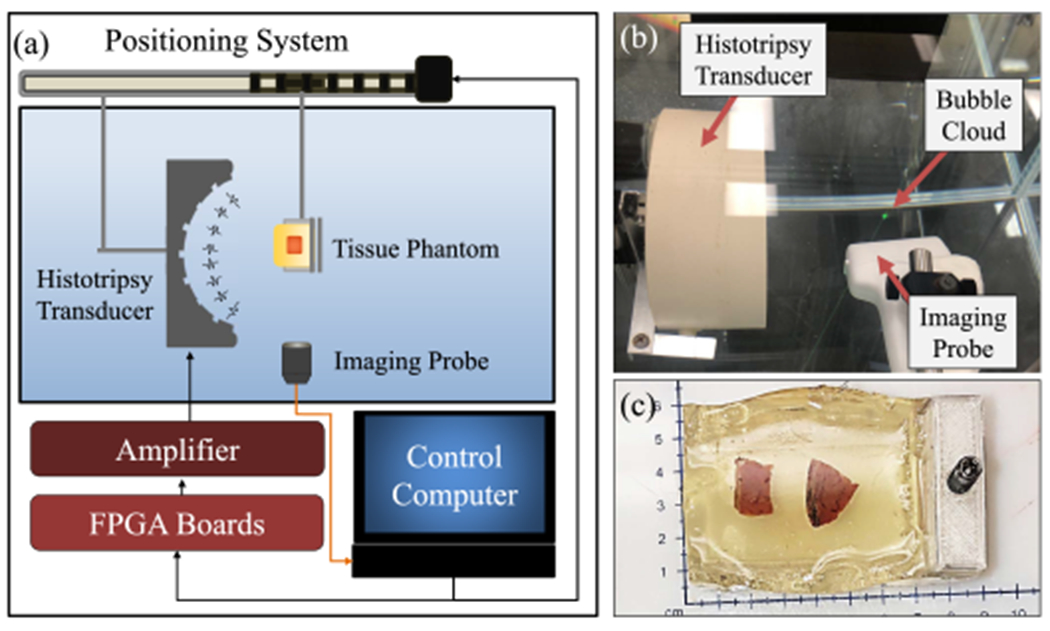
*Ex vivo* tissue ablation setup. (a) and (b) Histotripsy dose experiments were conducted using a 700-kHz transducer with the focus aligned in the center of the targeted tissue, with guidance from ultrasound imaging aligned orthogonally. (c) Tissues were embedded within the center of a gelatin phantom.

Liver tumor specimens were obtained by consent from patients with resectable HCC, colorectal LM (CLM), or CC tumors in accordance with the Institutional IRB approved protocol (Carilion Clinic, Roanoke Memorial Hospital, Roanoke, VA, USA). Patients with either biopsy-proven or radiologically diagnosed liver tumors were consented and enrolled in the study. At the time of resection, the resected tissues were sent fresh to pathology for processing, and any tissue above what was needed for clinical evaluation was donated to this study. When possible, surrounding liver parenchyma (LP) was also collected and used to evaluate the histotripsy doses for ablating LP tissue within the surgical margin. After harvesting, the tumor specimens were placed in a 0.9% degassed saline solution. Before treatment, samples were degassed inside a saline solution in a vacuum chamber (20–25 kPa) for 30–60 min. After degassing, the harvested tumor samples were sectioned into 0.5–1-cm^3^ samples and embedded in 7.5% degassed gelatin (porcine gelatin, Millipore Sigma) tissue phantoms for treatment [see Fig. 2(c)]. Gel phantoms containing the embedded tumor samples were then refrigerated for up to 8 h, with all specimens treated within 24 h of the initial harvest.

### E. Histotripsy Ex Vivo Ablation Procedure

The treatment doses that required ablating HCC, CLM, and CC tumors were investigated by histotripsy applying to the excised tissue samples embedded inside gelatin phantoms, as described above (see Fig. 2). For all experiments, the 700-kHz histotripsy therapy transducer was used to apply five-cycle pulses to the center of the tissue samples at a PRF of 500 Hz. These parameters closely match the histotripsy parameters used in previous shock-scattering histotripsy liver ablation studies [15], [16], [18], [27]. For each treatment, tissue samples embedded in gelatin were mounted to a motorized 3-D positioning system and aligned to the focus of the therapy transducer (see Fig. 2). To guide treatments and visualize the histotripsy cavitation bubble cloud in real time, an ultrasound imaging probe (SL18-5, AIXPLORER, SuperSonic Imagine) was mounted perpendicularly to the tissue and aligned to the focal location of the therapy transducer [see Fig. 2(b)]. The focus of the histotripsy transducer was located and marked on the imaging screen before each experiment by generating a bubble cloud in open water and then marking the location on the imaging screen before aligning the tissue at the focus. Tissue samples were then positioned such that the focal point of the transducer was located at the desired location within the tissue.

The pressure required to generate histotripsy cavitation inside each tissue sample was measured by gradually increasing the pressure at the focus until a cavitation cloud was identified on US imaging. The pressure at which a consistent histotripsy bubble cloud was generated inside each tissue sample was recorded as the cavitation cloud threshold before conducting volumetric ablation experiments in each sample.

Histotripsy was applied to the targeted volume at a pressure level chosen to be ~30% above the cavitation threshold, which corresponded to treatments with applied *p*-between 19 and 33 MPa, depending upon the sample. Volumetric treatments were then applied automatically to a predetermined 3-D grid of equally spaced treatment points, with 2.5-mm spacing in the axial direction and 1-mm spacing in the later directions to ensure overlap between adjacent bubble clouds. For each experiment, this grid of points was treated with histotripsy by mechanically scanning the therapy focal zone to cover the targeted portion of the tumor sample. The ideal treatment was a 5 mm × 5 mm × 5 mm cube consisting of 75 total points to dwell at. Given that some samples were too small to achieve this with a contained ablation volume, not all ablations were this large. The desired size for each sample was 15 mm × 15 mm × 15 mm. However, due to the irregular shapes of the tumors and the small size of some samples, this was not always achievable. The smallest sample treated in this study was a CC tumor that was approximately 8 mm × 8 mm × 8 mm, which led to a treatment size of 2.5 mm × 3 mm × 3 mm.

For each tumor type, three to five groups were treated with histotripsy at different doses to characterize the extent of tissue ablation generated at different histotripsy doses (i.e., the number of pulses/point). The number of pulses applied per point ranged from 500 to 2000 pulses/point for HCC and CLM tumors and 1000 to 4000 pulses/point for CC tumors. These treatment doses were chosen based on previous liver histotripsy studies [15], [24], [40], with the higher doses used for CC tumors based upon a combination of previous histotripsy studies on excised tissues [30], the relationship between histotripsy susceptibility and mechanical properties [24], [30], and the literature showing that CC has an increased Young’s modulus [32]. In addition to the liver tumor ablation experiments, a separate set of treatments was conducted on LP tissues collected from the surgical margins around the tumors at doses of 250–1500 pulses/point. A sample size of *n* = 3 per tissue type and treatment dose was used for all experiments when possible. However, due to limitations in tissue availability, this sample size could not be achieved for all tissues and all doses. Based on the availability of donated tissue samples, a total of 49 experimental treatments were conducted in this study, including eight HCC tumor samples, 15 CLM tumor samples, 12 CC tumor samples, and nine LP tissue samples. For each tissue, the sample numbers treated per dose are listed in Table I.

### F. Histology & Morphological Analysis

After treatment, tissue specimens were visually inspected and fixed in 10% formalin for at least 24 h before sectioning and staining. Given that each treatment was focused on the center portion of the tissue, the central region of each tissue was sectioned into a cassette. Using standard protocols, all tissues were paraffin-embedded and stained using hematoxylin and eosin (H&E), by ViTALS (Virginia Tech Animal Laboratory Services, Blacksburg, VA, USA), to assess for histotripsy damage to the tissue parenchyma and any other changes in structure and density of collagen and other tissue structures within the ablation volume. To determine the estimated extent of tissue ablated within the targeted volume, regions of cellular damage were compared with regions that were outside of the ablation zone. Results were then verified by a veterinary pathologist (SCO) and a human pathologist (DG) to ensure accuracy and avoid biases. Histology images are representative of the samples treated.

## III. Results

### A. Histotripsy Ablation of Subcutaneous PDX CC Tumors

**Fig. 3.**
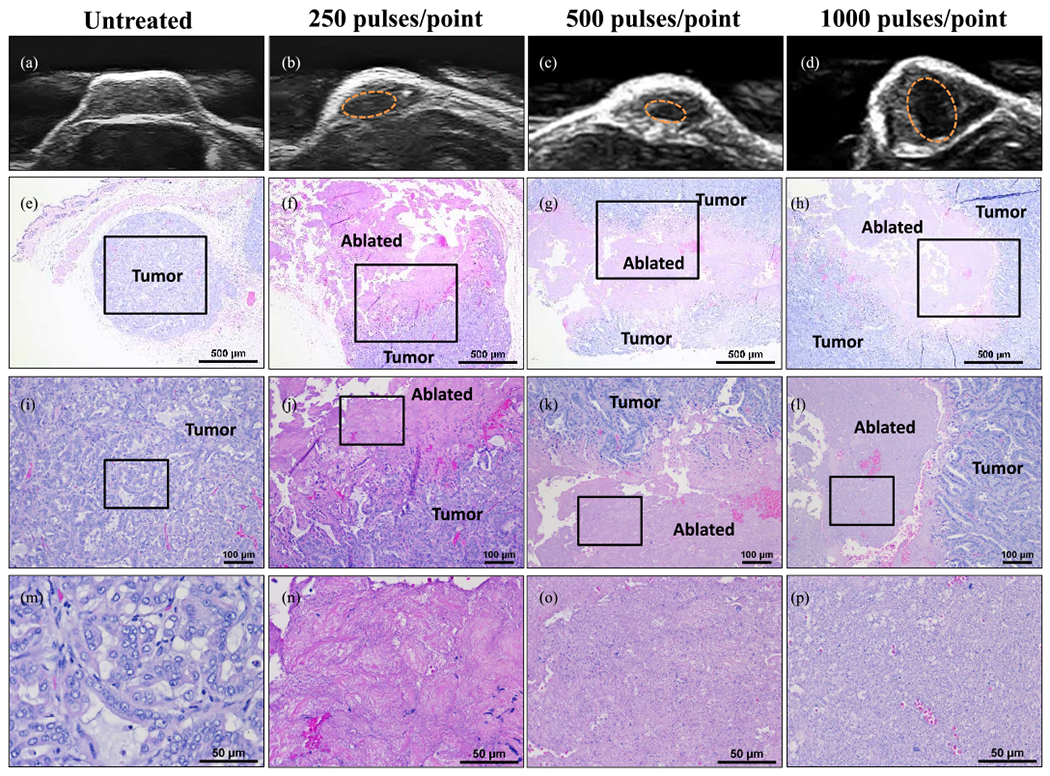
Dotted orange circles on US images. (a)–(d) Approximate region of ablation. (e)–(h) Corresponding to ablation region on 4× H&E. (j)–(l) 10x magnification H&E images of control and ablated region. (m)–(p) 40× magnification H&E images show ablation margins. Black boxes indicate area magnified in respective images. Scale bar on (e)–(h) 4× H&E images = 500 *μ*m, (j)–(l) 10× scale bar = 100 *μ*m, and (m)–(p) 40× scale bar = 50 *μ*m.

Well-defined histotripsy bubble clouds were observed at the focal locations in all tumors at the beginning of the treatment [see Fig. 1(b)]. Throughout the automated volumetric treatments, the bubble clouds were continuously observed at the focus as dynamically changing hyperechoic regions within the tissue. After the treatment, histotripsy ablation zones that matched the regions exposed to the bubble cloud could be visualized on ultrasound imaging at each of the three doses tested, with more clearly defined hypoechoic regions observed on ultrasound imaging as the treatment dose increased from 250 to 1000 pulses/point [see Fig. 3(b)–(d)]. The histological analysis of the ablation zones showed complete ablation (no remaining intact cells) of the CC tumors within the center of the treated region after 250-pulses/point treatments [see Fig. 3(j)]. Similarly, results for the 500- and 1000-pulses/point treatment groups showed complete ablation of the CC tumors inside the treated regions [see Fig. 3(k) and (l)]. The histological analysis also showed more well-defined treatment margins with increasing treatment dose [see Fig. 3(f)–(h)]. More specifically, results from the 250- and 500-pulses/point treatments showed small (<150 *μ*m) regions of disrupted tissue on the margins between ablated and intact tissue that included intact cells within the boundary region [see Fig. 3(f) and (g)]. In contrast, these partially treated boundary regions were not observed for the 1000-pulses/point treatments that contained a well-defined boundary between the completely treated region and the surrounding intact tissue [see Fig. 3(h)].

### B. Histotripsy Bubble Cloud Generation in Ex Vivo Tissue Specimens

The *p*-required to generate histotripsy bubble clouds inside each of the different tissue types using the 700-kHz clinical histotripsy transducer was measured to be 22.2 ± 1.2, 17.7 ± 3.1, 16.5 ± 2.6, and 20.6 + 4.6 MPa for HCC, CLM, CC, and LP samples, respectively. In general, the bubble clouds in all tissue types showed typical histotripsy features with a dense dynamically changing hyperechoic bubble cloud observed within or near the focal region of the transducer, as visualized on US imaging. During volumetric ablation experiments, the hyperechoic bubble clouds were maintained throughout the treatments in all tissue experiments. In some samples, particularly, samples smaller than the desired 15 mm × 15 mm × 15 mm dimensions, the cavitation bubble clouds formed at the interface of the gelatin phantom and the tissue including instances of both prefocal and postfocal cavitation clouds. These effects were observed more often in the CLM and CC samples. In cases in which prefocal cavitation was noted during the initial bubble cloud generation, the treatment zone was adjusted to a deeper in the sample when possible and the volumetric treatments were only included as part of this study for samples in which a focal bubble cloud remained present for the duration of the treatment.

### C. Histotripsy Ablation of Ex Vivo HCC Specimens

**Fig. 4.**
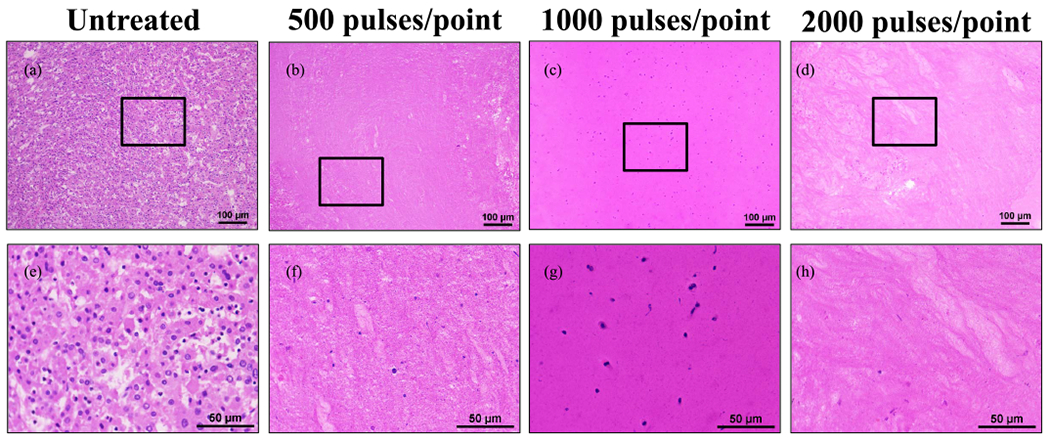
Comparison of untreated human HCC tumors with *ex vivo* ablations at 500, 1000, and 2000 pulses/point. (a)–(d) 10× magnification scale bar = 100 *μ*m. (e)–(h) 40× scale bar = 50 *μ*m. Black boxes on 10× images indicate area magnified in respective 40× images. Histology images are representative of the samples treated.

For HCC samples treated with 500 or more pulses/point [see Fig. 4(b)–(d) and (f) and (h)], histotripsy resulted in complete ablation of the targeted tumor volume with no notable regions of intact cells remaining inside the ablated area. There was tumor necrosis characterized by remaining cell nuclei and other subcellular tissue debris inside the treated regions on these samples, with the number of these nuclei reduced in concentration for the higher treatment doses. At 1000 pulses/point or more, there was a complete loss of cellular structures characterized as tissue ablated to amorphous debris.

### D. Histotripsy Ablation of Ex Vivo CLM Specimens

**Fig. 5.**
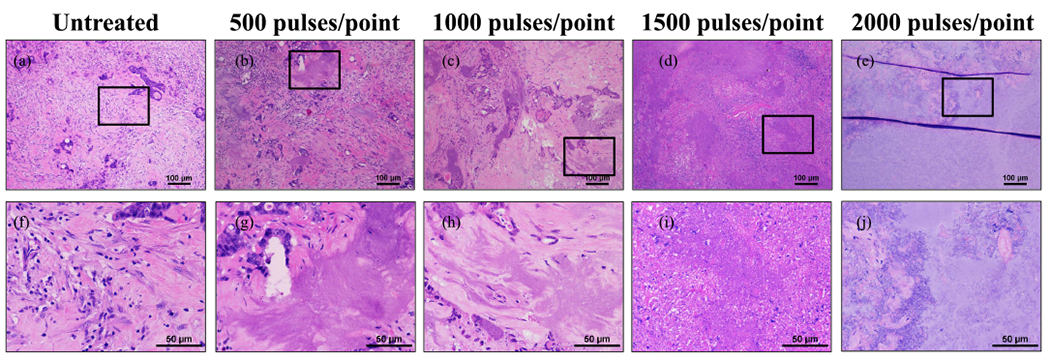
Comparison of untreated human CLM tumors with those treated at 500, 1000, 1500, and 2000 pulses/point. (a)–(e) 10× magnification scale bar = 100 *μ*m. (f)–(j) 40× scale bar = 50 *μ*m. Black boxes on 10× images indicate area magnified in respective 40× image. Histology images are representative of the samples treated.

The histological analysis demonstrated partial tumor ablation for CLM samples treated with 500 pulses/point, with more than 75% of cells within the treated region remaining after treatment [see Fig. 5(b) and (g)]. Small, sporadic foci of ablation were observed inside of these samples, with the majority of the treatment volume remaining intact. For 1000-pulses/point CLM samples, a larger portion of the tumor samples was observed to be ablated after treatment with ~25%–75% of the targeted region containing viable tumor tissue after histotripsy [see Fig. 5(c) and (h)]. At the higher treatment doses of 1500 and 2000 pulses/point, histotripsy was observed to generate significantly more tissue damage with complete ablation of the targeted tumor regions observed in some of the samples at both doses [see Fig. 5(d), (e), (i), and (j)]. However, results showed a large amount of variability in the extent of ablation among treatments within each group, with some samples showing only partial tumor ablation. More specifically, the number of viable cells remaining after treatment ranged from <5% (complete ablation) to ~25% in each group, with complete ablation achieved in two of the three samples at 1500 pulses/point and one of the two samples tested at 2000 pulses/point. It was noted that the tissues that did not show complete ablation within these groups appeared to contain more glandular structures and, thus, were well-differentiated. Less differentiated tumors with the poor cellular organization had greater ablation. Similarly, the histological analysis showed the CLM tumors tested in this study exhibited varying degrees of fibrosis within the tumor. Partial ablations were most often exhibited by CLM tumors with a robust, desmoplastic (or fibrous) response. For example, for the 1000-pulses/point samples shown in Fig. 5, the ablated regions of the tumor are found sporadically within the more fibrous regions of the tissue showing loss of cellular detail but still maintaining the fibrotic tissue architecture [see Fig. 5(c) and (h)]. In contrast, the extent of ablation was significantly higher within the less fibrotic regions of these tumors. This effect can be seen in the example 2000-pulses/point image shown in Fig. 5, in which large regions of the tissue were fully ablated, with the remaining tissue lacking cellular detail but with a few nuclei corresponding to the more fibrotic portions of the tumor.

### E. Histotripsy Ablation of Ex Vivo CC Specimens

**Fig. 6.**
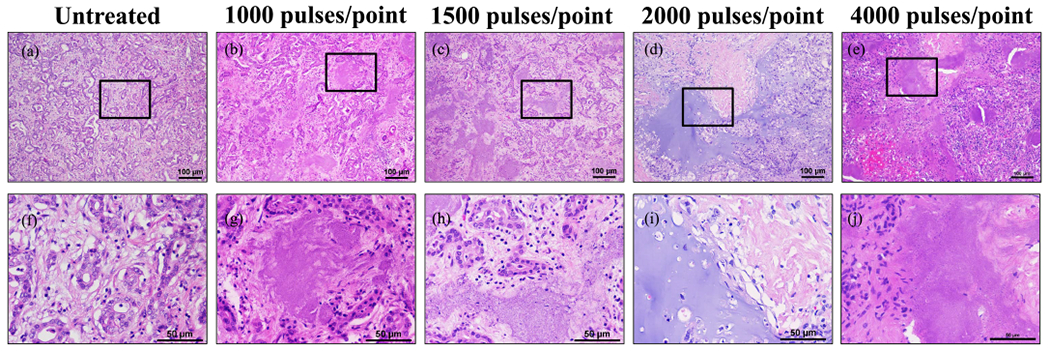
Comparison of untreated human CC tumors with those treated at 1000, 1500, 2000, and 4000 pulses/point. (a)–(e) 10× magnification scale bar = 100 *μ*m. (f)–(j) 40× scale bar = 50 *μ*m. Black boxes on 10× images indicate area magnified in respective 40× image. Histology images are representative of the samples treated.

The histological analysis demonstrated partial tumor ablation for CC samples treated with 1000 pulses/point, with sporadic foci of ablated tumor observed throughout the treated volume with interspersed regions of intact tumor [see Fig. 6(b) and (g)]. Results for the 1500- and 2000-pulses/point treatments showed similar results with larger regions of complete loss of cellular detail, supporting a completely ablated portion of the tumor in these samples (see Fig. 6) compared to the 1000-pulses/point group [see Fig. 6(b) and (g)]. For example, the 2000-pulses/point sample shown in Fig. 6 has two visible regions of the tumor, which were not fully ablated; both of them correspond to more fibrotic regions of the tissue. In contrast, the fully ablated regions within this sample appear to correspond to less dense and less fibrotic regions of the tumor. At the highest treatment dose of 4000 pulses/point, histotripsy again resulted in a similar result with only partial ablation generating within each of the CC tumors and ~50%–75% of the cells showing viable nuclei but with loss of cytoplasmic detail within the ablation region remaining after treatment [see Fig. 6(e) and (j)]. In these samples, the sporadic foci of ablation again appeared to be located in the regions of the tissue low in fibrosis (see Fig. 6). Looking at these foci under higher magnification, the tissue within these ablated regions showed complete ablation with no remaining viable cells. In contrast, the less ablated regions of the CC tumors were highly more fibrotic with >75%–90% of viable cells remaining after treatment, as shown in Fig. S1 in the Supplementary Material. Finally, it is important to note that a high amount of variability in the extent of ablation was observed among treatments within each group. Several of the treatments resulted in >75% ablation (<25% of viable cells remaining) at dosing regimens of 1000, 1500, 2000, and 4000 pulses/point, whereas other treatments resulted in <25% ablation with the majority of the targeted region remaining intact after treatment.

### F. Histotripsy Ablation of Ex Vivo LP Specimens

**Fig. 7.**
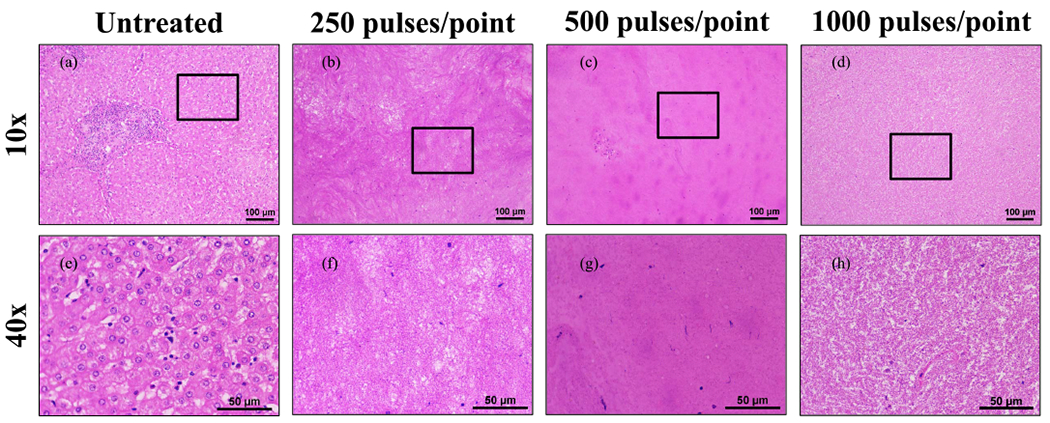
Nonsteatotic LP treatment at 250, 500, and 1000 pulses/point demonstrating less uniform ablations. (a)–(d) 10× magnification scale bar = 100 *μ*m. (e)–(h) 40× scale bar = 50 *μ*m. Black boxes on 10× images indicate area magnified in respective 40× images. Histology images are representative of the samples treated.

**Fig. 8.**
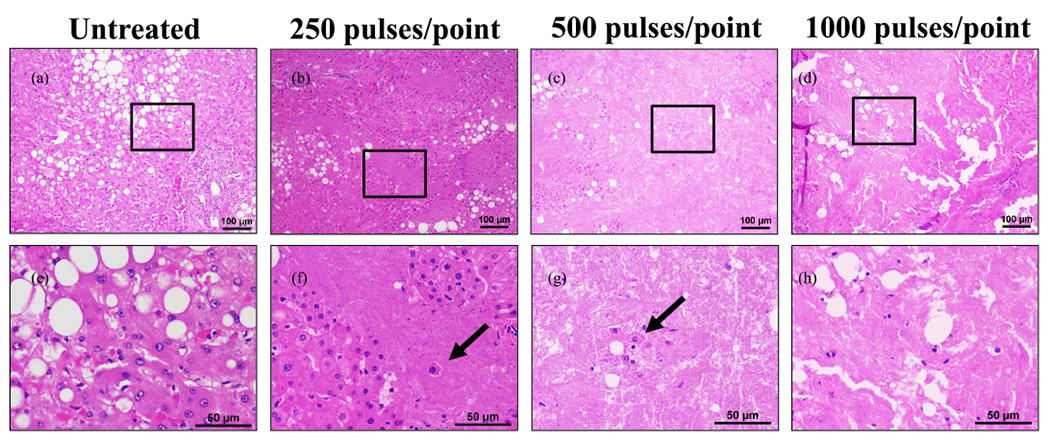
Steatotic LP treatment at 250, 500, and 1000 pulses/point demonstrating less uniform ablations. (a)–(d) 10× magnification scale bar = 100 *μ*m. (e)–(h) 40× scale bar = 50 *μ*m. Black boxes on 10× images indicate area magnified in respective 40× images. Arrows indicate example cells within an ablated region that appear to be intact. Histology images are representative of the samples treated.

Part of the surrounding LP was excised in addition to the tumor for six patients enrolled in this study. Four patients had hepatic steatosis as identified by abdominal imaging. One patient had hemochromatosis in addition to steatosis, one was hepatitis C positive with fibrosis, and another patient had no identifiable liver disease. These samples were divided based on the presence of steatosis. On histology, tissues from patients with hepatic steatosis were readily apparent based on the presence of macrovesicular fat globules within the tissues. For nonsteatotic samples, a sample size of *n* = 1 was conducted at 250, 500, and 1000 pulses/point. Steatotic samples were treated at *n* = 2 at 250 pulses/point, *n* = 3 for 500 pulses/point, and *n* = 1 for 1000 pulses/point. Complete ablation was formed within the tissues that did not have hepatic steatosis even at doses as low as 250 pulses/point (see Fig. 7). In comparison, tissues with hepatic steatosis were noted to have sporadic foci of ablations at 250 pulses/point with more complete ablation generated at the higher doses. For instance, only a few remaining viable cells were visible inside the ablation zone after 500 pulses/point, and complete ablation of the treated region was observed after 1000 pulses/point (see Fig. 8).

## IV. Discussion

To study the potential of histotripsy for CC tumor ablation, this work first investigated the *in vivo* feasibility of histotripsy CC ablation in a PDX mouse model, with results showing that histotripsy could achieve precise and complete ablation within the targeted region of the tumor. The treatment doses used to treat the CC tumors in this study were higher than those reported previously for xenografted HCC tumors [19]. Given the known correlation between tissue mechanical properties and susceptibility to histotripsy, we hypothesize that this difference is likely due to the higher mechanical strength of CC tumors [24], [30], [31]. Overall, the results from this *in vivo* feasibility study suggest that histotripsy will be capable of treating CC tumors if the proper treatment dose is applied.

In the second part of this study, the effectiveness of histotripsy for ablating CC tumors was compared to HCC and CLM tumors, as well as LP tissue from the surgical margin, in *ex vivo* experiments using human tumor specimens. Overall, the results of these experiments help to demonstrate the histotripsy dosimetry metrics for treating CC in comparison to HCC and CLM liver tumors. More specifically, the results showed that both the CC and CLM tumors required higher treatment doses before tissue ablation was observed in comparison to the softer HCC and LP samples that showed complete tumor ablation at lower treatment doses. These findings match prior studies that have shown fibrous tissues, which have been reported to have higher mechanical strength, are more resistant to histotripsy, and, therefore, require higher treatment doses [24], [30], [31]. Results from HCC treatments demonstrated that HCC can be completely ablated with histotripsy of fewer than 500 pulses/point, agreeing with previous reports that histotripsy has the potential for rapid, precise, and complete ablation of primary liver tumors. Since CLM tumors are stiffer than HCC tumors [30], [24], we hypothesized that higher treatment doses would be required for generating ablation in these samples. Results supported this hypothesis, showing that histotripsy was able to achieve only small regions of partial ablation for 500 pulses/point. At higher treatment doses of 1500 and 2000 pulses/point, nearly complete or complete ablation was observed in several CLM samples, with the only untreated regions corresponding to highly fibrous regions within the tumor sample.

For *ex vivo* experiments with CC tumors, the trends were similar to those observed for CLM with higher doses again being needed to generate ablation in the CC tumors. Even at the highest doses treated in this study (4000 pulses/point), there were no cases in which we observed complete ablation of the entire treatment volume within the CC tumors, suggesting that higher doses will be needed to achieve full ablation of these highly fibrous CC tumors. This finding appeared to be at least partially due to the large variation in tissue composition between samples and the heterogeneity of the tissue within a given sample (see Fig. S1 in the Supplementary Material). Partial ablations were more pronounced in the CC and CLM tumor samples. We hypothesize that this is due to the higher mechanical strength and the more fibrous nature of these tumors.

Although complete ablation was not generated in the *ex vivo* CC tumors, significant ablation was observed for treatments at the two highest doses tested, with nearly 75% of the treated volume ablated after 2000 and 4000 pulses/point. Overall, these results highlight the need for tumor-specific treatment strategies in future clinical treatments of liver tumors with histotripsy to ensure complete ablation of CLM and CC tumors and more efficient ablation of HCC tumors. For instance, these approaches could include the development of guidelines for the treatment doses (histotripsy pulses/point) needed for treating different tumor types and the development of optimized pulsing parameters although further work would be needed in order to investigate this possibility. In addition, improved real-time feedback methods could be utilized to ensure complete therapeutic ablation of specific tumors, such as those previously developed using bubble-induced color doppler feedback [41], [42] or cavitation detection [43].

In addition to the differences in ablation observed between tumor types, another important observation from this study was the variability in ablation between samples of a given tumor type. This result was possibly due in part to variations in the tissue composition, mainly fibrotic deposits, between any two tumors of the same type and the heterogeneity of the tissue within a single tumor. This effect would explain why the more uniform and softer HCC samples consistently resulted in complete ablation at lower treatment doses compared to more variable results and less complete ablation observed in the more heterogeneous and fibrous CLM and CC tumors. Looking at the regions of partial ablation in these samples, results showed that the foci of ablation interspersed within regions that were not ablated corresponded to the areas with less fibrosis. This result suggests that there is a need for ensuring that histotripsy treatment doses are either optimized for each patient based on pretreatment assessments of the tumor composition and mechanical characters, such as through MRI or ultrasound elastography. Alternatively, a sufficient treatment dose could be identified based on the most fibrous tumors within each type based upon historically determined mechanical properties. For example, it is possible that the different subtypes of intrahepatic CC (mass-forming, periductal, and intraductal) may respond differently to histotripsy both in the required treatment dose and the efficacy of histotripsy for achieving the desired clinical outcome. Future work should explore this possibility in order to establish the optimal role of histotripsy for the treatment of each subtype of intrahepatic CC, as well as extrahepatic CC, and determine if tumor type-specific treatment methods are required.

However, although we believe that the variations in tissue composition are likely to explain some of the variability in our *ex vivo* tissue experiments and should be considered in future clinical histotripsy treatments, it is also important to note that these findings could also be due to the limitations of our tissue collection methods in this study. Unfortunately, due to the requirements of the clinical pathologists before tissue collection, the samples collected for this study were often smaller than the desired size for performing optimal *ex vivo* histotripsy experiments. In these smaller samples, prefocal bubble clouds were more often observed on the tissue-gel interface especially in the more fibrous CLM and CC tumors, preventing a more uniform histotripsy treatment from being applied to the tissue volume and likely contributing to some of the variable ablation results observed in those tissues. These effects are not expected to occur *in vivo* where much larger tumors that are surrounded by adjacent LP will be treated with histotripsy, therefore minimizing the risk of prefocal cavitation that causes inconsistent tumor ablation.

Another observation from the *ex vivo* experiments conducted in this work was the finding that nonsteatotic LP was readily ablated with histotripsy, similar to HCC samples, whereas higher treatment doses were required to ablate steatotic LP samples. This finding is important for future liver cancer treatments with histotripsy since it is a standard clinical practice to ablate a margin of the surrounding liver around the tumor to destroy all microscopic foci of tumor cells that might be within these regions. The finding that the LP surrounding tumors in patients with hepatic steatosis, or fatty liver, required higher treatment doses compared to those without this disease, should be noted in future studies optimizing treatment parameters for hepatic histotripsy. It is also possible that fatty pockets dispersed throughout the liver could contribute to more diffuse or prefocal cavitation. This effect would make sense since previous work has shown a significantly lower cavitation threshold for fat compared to water-based parenchymal tissues [20], [21]. However, since no consistent differences in cavitation were observed between the steatotic and nonsteatotic LP samples treated in this study, further work remains needed to investigate the role that fat deposits play in hepatic histotripsy and whether any differences in the cavitation threshold can cause variation in ablation completeness similar to what has been observed for differences in tissue fibrosis.

## V. Conclusion

This study tested the potential of histotripsy for the treatment of CC tumors for the first time, with results suggesting that histotripsy has the potential to be used as a noninvasive ablation method for the treatment of CC. Results from the *in vivo* experiments demonstrated that histotripsy could generate precise and complete ablation of CC tumors in a PDX CC mouse model. In the second part of this work, which compared the histotripsy treatment dose required to ablate different types of liver tumors, results showed that CC and CLM tumors required significantly higher doses than HCC tumors. These findings highlight the need for tumor-specific treatment strategies for future histotripsy liver cancer procedures to ensure that more fibrous tumor types, as well as more fibrous regions within a tumor, are completely ablated during histotripsy. Overall, the findings of this study support further investigation of histotripsy for the treatment of CC in addition to HCC and CLM.

## Supplementary Material

Supplementary Material

## Figures and Tables

**TABLE I:** *Ex Vivo* Sample Numbers by Tissue Type and Dose

	250	500	1000	1500	2000	4000
***HCC***		n=2	n=2		n=1	
***CLM***		n=3	n=3	n=3		
***CC***			n=3	n=3	n=3	n=2
***LP***	n=3	n=4	n=2			
