# Supplementary figures and images for: Histotripsy for the Treatment of Cholangiocarcinoma Liver Tumors: In Vivo Feasibility and Ex Vivo Dosimetry Study
Source: IEEE Trans Ultrason Ferroelectr Freq Control. Author manuscript; Available in PMC 2022 Jul 20. (PMC9297335; doi:10.1109/TUFFC.2021.3073563)

Supplemental Figure S1

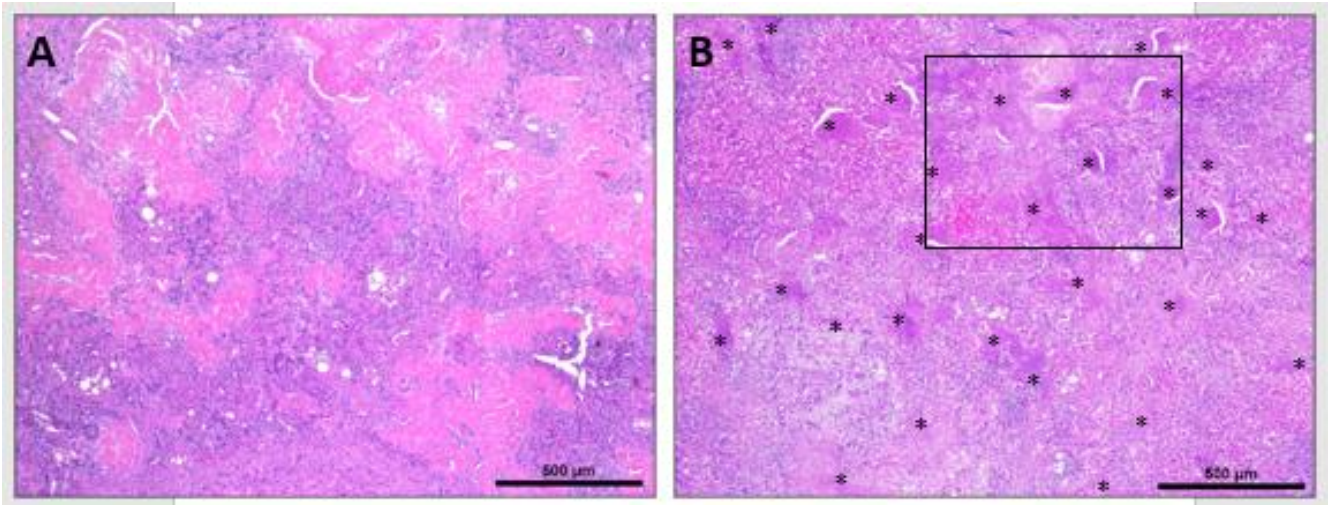

Supplement: Supplementary Material [file NIHMS1821487-supplement-Supplementary_Material.pdf]
